# HABIT-ILE@home for Children With Bilateral Cerebral Palsy: Protocol for Noninferiority and Superiority Randomized Controlled Trials

**DOI:** 10.2196/89872

**Published:** 2026-08-11

**Authors:** Zélie Rosselli, Merlin Somville, Edouard Ducoffre, Carlyne Arnould, Yannick Bleyenheuft, Geoffroy Saussez

**Affiliations:** 1 MSL-IN lab, Institute of Neuroscience, UCLouvain, Bruxelles, Belgium; 2 FfH Lab Department of Health and Medical Technologies, CeREF-Santé, Haute École Louvain en Hainaut, Rue Trieu Kaisin 136, Montigny-sur-Sambre, Wallonia, Belgium, +32488 80 81 34

**Keywords:** bilateral cerebral palsy, intensive intervention, motor function, HABIT-ILE, home-based training, telerehabilitation, Hand-Arm Bimanual Intensive Therapy Including Lower Extremities

## Abstract

**Background:**

Intensive motor learning interventions, such as Hand-Arm Bimanual Intensive Therapy Including Lower Extremities (HABIT-ILE), have demonstrated significant improvements in upper and lower extremity functions, as well as in bimanual performance and activities of daily living in children with cerebral palsy. However, the typical delivery of HABIT-ILE as a 2-week on-site camp can be inaccessible to families living in remote areas or with travel difficulties. Offering HABIT-ILE as a home-based telerehabilitation program could overcome these barriers.

**Objective:**

The study aims to evaluate the effectiveness of this approach through 2 randomized controlled trials.

**Methods:**

A total of 44 children with bilateral cerebral palsy (aged 5‐18 years) will participate. HABIT-ILE@home will follow the principles of HABIT-ILE on-site but will be delivered at home with the involvement of caregivers and remote supervision by trained therapists. A dedicated virtual device will facilitate intervention delivery and remote monitoring (REAtouch Lite). The first randomized controlled trial (RCT), RCT1, will assess the noninferiority of high-dose HABIT-ILE@home vs on-site HABIT-ILE (65 h over 2 weeks, 6.5 h/d). The second RCT (RCT2) will assess the superiority of a HABIT-ILE@home follow-up program delivered over 9 weeks compared to a nonspecific home follow-up (45 h in total, 5 h/wk). The Gross Motor Function Measure-66 (GMFM-66) is the primary outcome. Secondary outcomes include feasibility and adherence questionnaires, motor and functional outcome measures, as well as activities of daily living questionnaires, self-esteem, participation in life situations, and neuroimaging. Evaluations will be conducted before (T0) and after (T1) the 2 weeks of therapy, followed by a final assessment after the follow-up (T2).

**Results:**

Recruitment was completed in June 2024. All interventions were performed by November 2024. Data analysis was completed in May 2025.

**Conclusions:**

The results will first confirm or refute the noninferior efficacy of a telerehabilitation HABIT-ILE@home program compared to its typical on-site delivery. Second, the results will determine the added value of a specific HABIT-ILE@home follow-up after such high-dosage motor learning–based interventions.

## Introduction

### Background

Cerebral palsy (CP) represents the most prevalent cause of physical disability in the pediatric population, affecting 1 to nearly 4 children in 1000 newborns worldwide [1,2]. This major public health problem, caused by abnormalities in or damage to brain development, results in a range of symptoms that vary widely from child to child. While all children with CP will develop motor symptoms such as postural and movement control deficits, some will also develop additional nonmotor symptoms, including pain (75%), intellectual deficits (50%), language disorders (25%), epilepsy (25%), and behavioral and sleep disorders (20%‐25%) [3]. The consequences of these symptoms may vary in severity but are typically manifested as long-term functional limitations in daily activities, including dressing, eating, and personal hygiene [4].

To enhance individual motor function and functional performance, intensive therapies based on motor skill learning (MSL) have been demonstrated to be particularly efficacious [5]. Among these interventions, Hand-Arm Bimanual Intensive Therapy including Lower Extremities (HABIT-ILE) has been developed over the last decade with the purpose to simultaneously engage upper and lower extremities as well as trunk control through bimanual manipulative activities performed while sitting on a ball, standing, or walking [6]. HABIT-ILE has led to notable improvements in upper and lower extremity functions, as well as in bimanual performance and activities of daily living for children with bilateral and unilateral CP [6,7]. A previous study [8] has shown that upper extremity improvement was not attenuated by the simultaneous engagement of both the upper and lower extremities compared to a similar intervention solely focusing on the upper extremities. In addition, the HABIT-ILE approach has been demonstrated to facilitate significant enhancements in social participation and a reduction in mirror movements among children with unilateral CP [9]. The motor and functional improvements observed following HABIT-ILE seem to be linked to changes in cerebral activity, as evidenced by changes in the fronto-parietal and cerebellum networks, as well as in the integrity of corticospinal tract fibers [10,11]. The intervention is typically delivered as a 2-week rehabilitation camp, comprising 50 to 90 hours of on-site activities [12-14].

However, this mode of delivery raises limitations in terms of accessibility and scalability. The implementation of a 2-week day camp necessitates considerable commitment from families, who frequently must travel to reach the therapy site. As the number of locations offering HABIT-ILE therapy is currently limited, families who live a long way from the facility and who are unable to travel cannot benefit from this therapy. Moreover, the organization of such intensive therapies continues to represent a significant challenge, given the necessity for a relatively spacious training room, a substantial quantity of equipment, and the involvement of trained therapists [15]. To address these concerns, the introduction of a home-based HABIT-ILE telerehabilitation program combined with caregiver coaching may represent an alternative solution. Virtual devices may be an effective means of implementing MSL principles and facilitating remote communication with families [16]. The range of telerehabilitation devices is vast, from classic video games [17] to devices specifically designed for rehabilitation purposes [18-20]. Although not all devices appear equally effective in implementing all principles of MSL during virtual-based-interventions, these principles can be applied with specifically-designed devices and the supervision of a trained therapist [16].

Besides the accessibility and organizational limitations related to intensive interventions, no specific follow-up intervention has so far been provided to participants at the end of a HABIT-ILE camp to facilitate the transfer of skills at home [6-8]. Maintaining the skills acquired during the 2-week intervention requires daily practice to avoid an unfavorable motor cortical reorganization and a loss of the newly developed skill [21,22]. As such interventions are frequently conducted outside conventional health care settings that oversee children throughout the year, transmission of information might sometimes be challenging to ensure continuity of care for children [21]. Accordingly, it may be beneficial to investigate the efficacy of a specific low- to moderate-dosage HABIT-ILE home-based follow-up program delivered after a 2-week HABIT-ILE therapy to increase the possibility to transfer the newly learned abilities at home and promote further improvements.

### Aims and Hypotheses

Two randomized controlled trials (RCTs) are described in this protocol. The first RCT (RCT1) aims to evaluate whether a high-dosage HABIT-ILE telerehabilitation intervention (HABIT-ILE@home) can achieve improvements in motor and functional abilities, as well as in social participation and neuroplastic changes comparable to those obtained with a conventional high-dosage HABIT-ILE on-site camp in children with bilateral CP aged 5-18 years. Gross motor function, assessed using the Gross Motor Function Measure-66 (GMFM-66), will constitute the primary outcome measure. We hypothesize that significant improvements in gross motor function will be observed in both groups after the 2-week intervention and that improvements achieved following HABIT-ILE@home will be noninferior to those observed after the HABIT-ILE on-site camp.

The second RCT (RCT2) aims to determine whether a structured low- to moderate-dosage HABIT-ILE@home follow-up program provided over 9 weeks leads to greater retention and further improvement in motor and functional abilities as well as in social participation, compared to a nonspecific home follow-up intervention based on usual clinical practice following the intensive HABIT-ILE intervention. Gross motor function assessed using the GMFM-66 will constitute the primary outcome measure. We hypothesize that children receiving the specific HABIT-ILE@home follow-up intervention will demonstrate greater retention and additional improvements in gross motor function compared with children receiving the nonspecific home follow-up intervention.

## Methods

### Study Design

**Figure 1.**
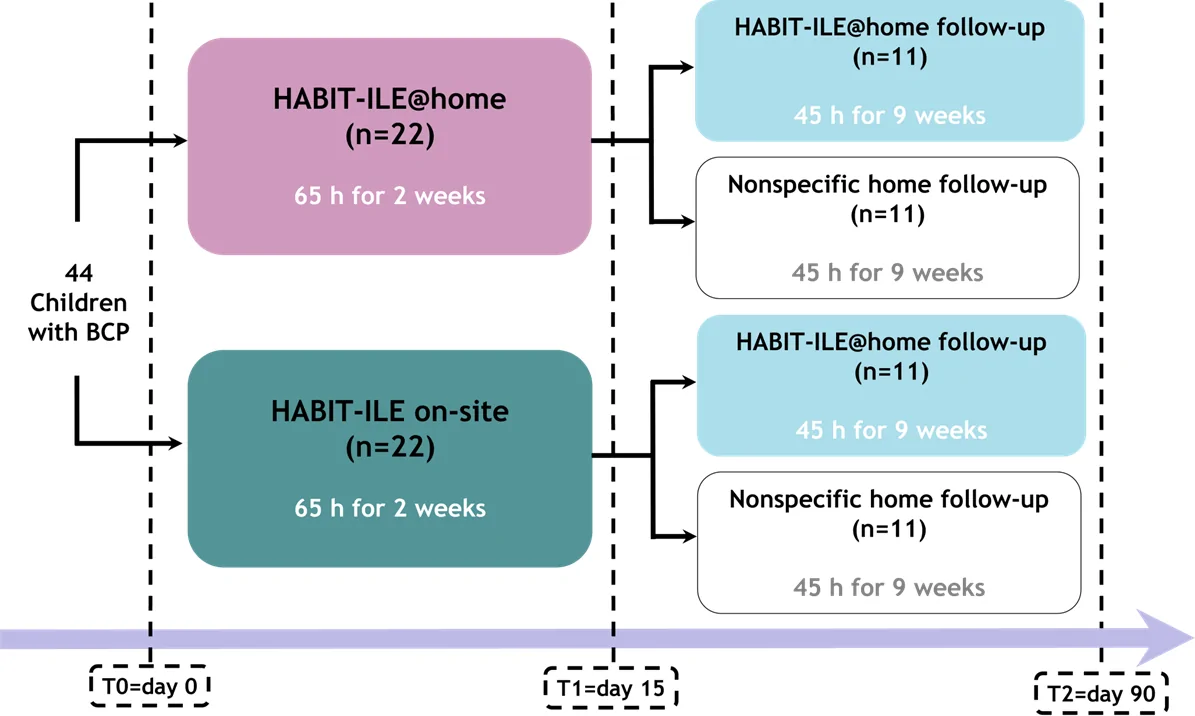
Study design for the implementation of Hand-Arm Bimanual Intensive Therapy Including Lower Extremities (HABIT-ILE) interventions in children with bilateral cerebral palsy (BCP). Forty-four children with BCP will be randomly assigned to either the HABIT-ILE@home group (n=22) or the HABIT-ILE on-site group (n=22). Both groups will complete an intensive 65-hour intervention over 2 weeks (T0-T1). Subsequently, each group will be further divided for a 9-week follow-up phase (T1-T2): half will continue with HABIT-ILE@home follow-up (n=11 per initial group), while the other half will engage in nonspecific follow-up home-based activities (n=11 per initial group). The timeline indicates assessment points at T0 (baseline), T1 (post intensive phase), and T2 (end of follow-up phase). h: hour; RCT: randomized controlled trial.

**Figure 2.**
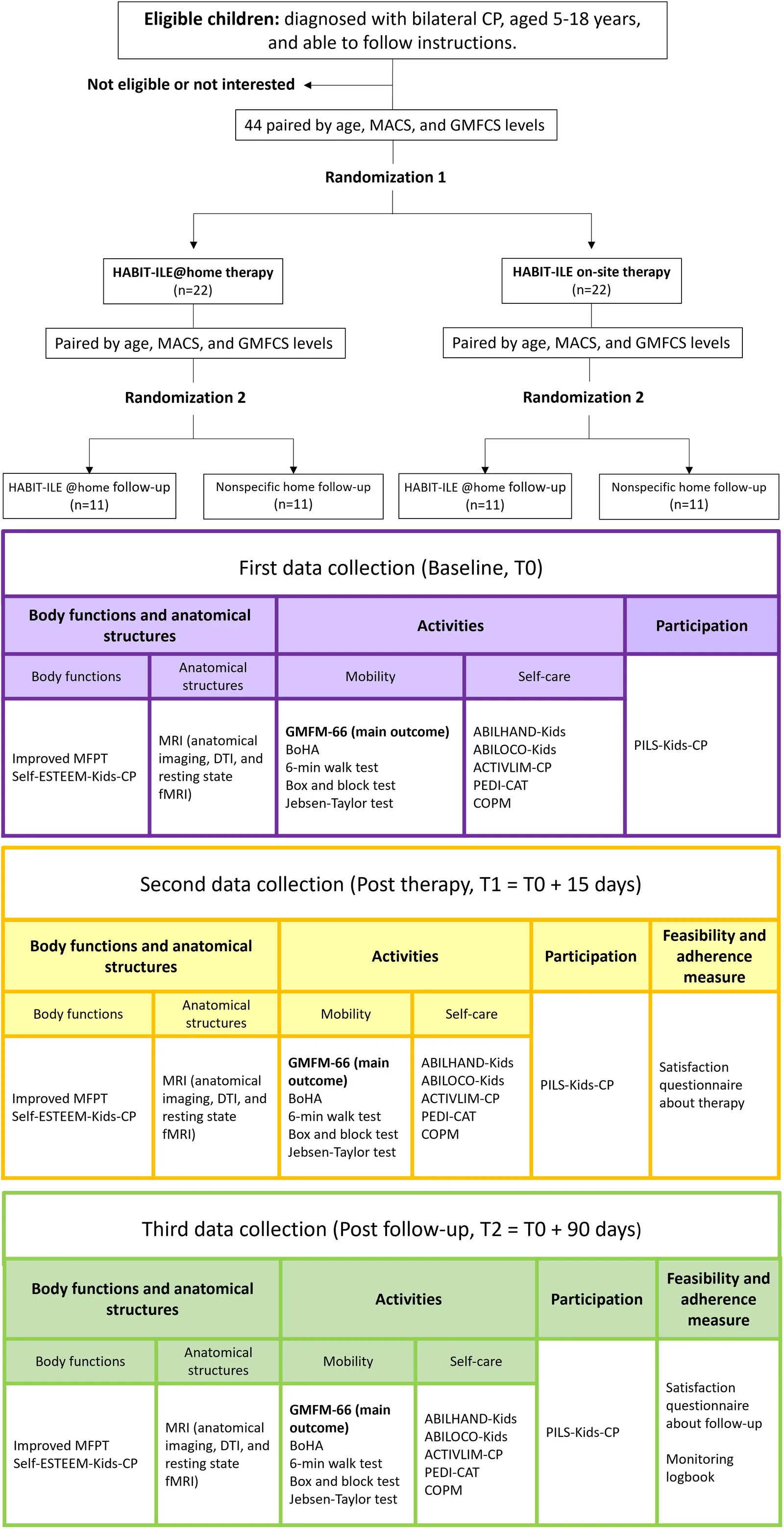
Study flowchart and data collection. Eligible children will be randomized into 2 groups: HABIT-ILE@home or Hand-Arm Bimanual Intensive Therapy Including Lower Extremities (HABIT-ILE) on-site therapy, followed by a second randomization into HABIT-ILE@home follow-up or nonspecific home follow-up subgroups. Data collection will occur at 3 time points: baseline (T0), post therapy (T1 = T0 + 15 days), and post follow-up (T2 = T0 + 90 days). Outcome measures will assess body functions, activities, participation, and feasibility across the study phases. BoHA: Both Hands Assessment; COPM: Canadian Occupational Performance Measure; CP: cerebral palsy; DTI: diffusion tensor imaging; fMRI: functional magnetic resonance imaging; GMFM-66: Gross Motor Function Measure-66; GMFCS: Gross Motor Function Classification System; MACS: Manual Ability Classification System; MFPT: Manual Form Perception Test; PEDI-CAT: Pediatric Evaluation of Disability Inventory–Computer Adaptive Test.

For RCT1, 44 participants will be randomized to 1 of the 2 groups (“HABIT-ILE@home” or “HABIT-ILE on-site camp,” 65 h for 2 wk), 22 children per group (Figure 1). The HABIT-ILE on-site camp will take place in Belgium with the presence of interventionists and a supervision team, whereas the HABIT-ILE@home intervention will take place at the children’s home with the constant presence of a caregiver (eg, parents, relatives, or other close individuals) and intermittent remote supervision. After this high-dosage camp, each group will then be further randomized into 2 subgroups of 11 participants (RCT2). One subgroup will receive the specific HABIT-ILE@home follow-up (5h/wk, for 9 wk), while the other will receive a nonspecific home follow-up based on their usual therapist’s recommendations. Recommendations of practice schedule given to caregivers will be similar for both follow-up groups: 5 hours per week at home for 9 weeks. Participants will be assessed before (T0) and after (T1) the 2 weeks of HABIT-ILE high-dosage camp, as well as at the end of the 9-week follow-up (T2). This study protocol is reported in accordance with the SPIRIT (Standard Protocol Items: Recommendations for Interventional Trials) guidelines (Figure 2).

### Recruitment

#### Participants

For this study, 44 children with bilateral CP aged 5-18 years will be included. Children will be recruited in Belgium and surrounding countries based on contacts with reference centers at Belgian hospitals or via spontaneous applications, notably following information on social networks.

#### Inclusion Criteria

Children will be considered eligible if they are aged 5-18 years, have a confirmed diagnosis of bilateral CP, and have sufficient cognitive skills to play simple games (ie, throwing a ball) and follow simple instructions. For the needs of the HABIT-ILE@home intervention program, a caregiver (ie, family member, relatives, and so on) must be available for 6.5 hours per day to accompany the child for the 2-week HABIT-ILE@home therapy and during 5 hours per week over the 9-week HABIT-ILE@home follow-up.

#### Exclusion Criteria

Children will not be eligible to participate if they present uncontrolled seizures, have had botulinum toxin injections, orthopedic surgery, or intensive therapy within the past 3 months, or if such interventions are planned during the study period, or if they present with severe visual or cognitive impairments interfering with treatment and/or assessments, or if they have any contraindications for magnetic resonance imaging (MRI).

### Randomization Process

At the end of the recruitment phase, children were paired by the investigators according to Manual Ability Classification System (MACS) level, Gross Motor Function Classification System (GMFCS) level, and age, in this order of priority. Within each pair, participants were randomly assigned a code number (1 or 2) using Research Randomizer. To ensure allocation concealment and limit selection bias, the correspondence between the code number and the intervention group was determined independently by a person from an affiliated laboratory who was not involved in recruitment, assessment, or intervention delivery. This person indicated whether code 1 corresponded to the control group or to the treatment group after the pairs and code numbers had been generated. For RCT2, participants within each group of RCT1 were randomized again according to the same procedure to either the HABIT-ILE@home follow-up or the nonspecific home follow-up group (Figure 1).

### Sample Size

#### RCT1: High-Dosage Intervention (2-Week Home vs On-Site Camp)

Sample size calculation was performed using PASS (version 14.0.15; NCSS) for a one-sided noninferiority *t* test comparing 2 independent means. The analysis was based on the mean improvement in GMFM-66 observed in the study of Bleyenheuft et al [7]. We hypothesized that there would be a true difference of Gross Motor Function Measure (GMFM) units between groups, with an α of .05 and a 1-*β* of 0.9. We estimate that the noninferiority margin (NIM) could reach a maximum of 2 GMFM units since this value is the minimal clinically important difference (MCID) of the GMFM-66. The data are drawn from populations with SDs of 1.9 GMFM units. Consequently, 17 participants are needed per group (N=34). Considering 25% potential dropouts per group, 44 participants will be included.

#### RCT2: Low- to Moderate-Dosage Home Follow-Up Interventions (9-Week HABIT-ILE@home Follow-Up vs Nonspecific Home Follow-Up)

For RCT2, sample size calculation was also performed using PASS (version 14.0.15; NCSS) for a 2-sided superiority *t* test comparing 2 independent means. The analysis was based on the mean improvement in GMFM-66 observed in the study of Bleyenheuft et al [7]. In that study of school-aged children, the duration of the intervention was 84.5 hours and the GMFM-66 increased by 7 GMFM units. We thus hypothesized that, for 45 hours of intervention, there would be a true difference of 3.75 GMFM units between groups, with an α of .05 and a 1-*β* of 0.8. We estimate that the superiority margin will be 2 GMFM units since this value is the MCID of the GMFM-66. The data are drawn from populations with a SD of 1.9 GMFM units. Consequently, 16 participants are needed per group (N=32). Considering 25% potential dropouts, 40 participants will be included.

### Current Status of the Protocol

This protocol was written in 2024, after the study methods had been finalized and recruitment and data collection completed, but before completion of the final statistical analyses.

### Blinding Procedure

External accredited or experienced raters, blinded to group allocation and timing of assessment, will score videos of the main outcome of this multicentric RCT (GMFM-66), as well as one of the secondary outcomes (Both Hands Assessment [BoHA]). Neuroimaging and accelerometers data analyses will also be conducted by raters blinded to group allocation and timing of assessment. Data will be anonymized (using allocation codes) and delivered for analysis after the RCT ends.

### Study Interventions

The intervention is described in accordance with the Template for the Intervention Description and Replication (TIDieR)-telehealth checklist, provided as in Checklist 1 [23].

#### RCT1: High-Dosage Intervention (2-Week Home vs On-Site Camp)

##### Overview

For RCT1, all children will take part in a high-dosage HABIT-ILE intervention, either in an on-site camp or at home with a telerehabilitation program. All children will benefit from 65 hours of therapy at a rate of 6.5 hours per day, 5 days per week, for 2 weeks.

##### HABIT-ILE On-Site Camp (Control Group)

**Figure 3.**
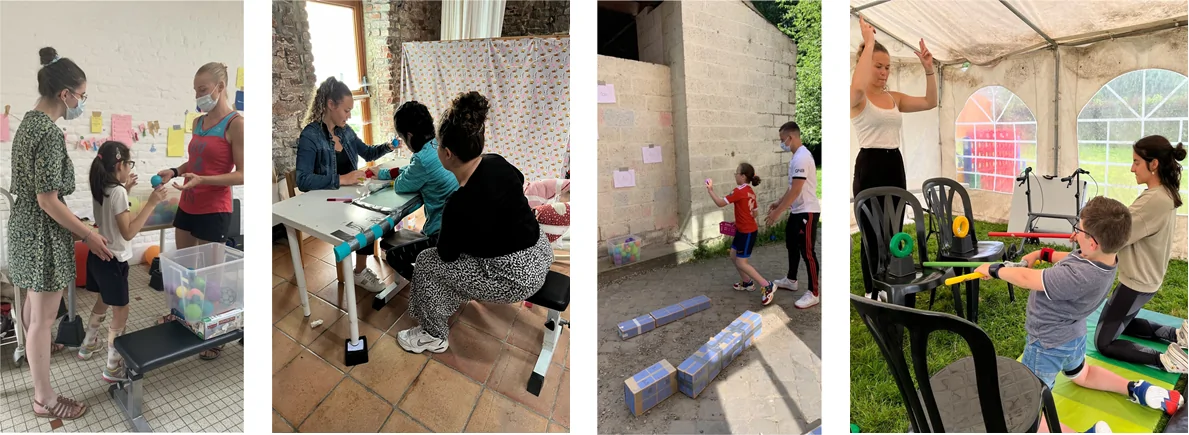
Activities Performed During an On-Site Hand-Arm Bimanual Intensive Therapy Including Lower Extremities (HABIT-ILE) Camp.

HABIT-ILE therapy for children with bilateral CP aims to improve motor control and coordination of both upper and lower extremities with trunk control in activities of daily living. It is delivered as a 2-week day-camp intervention where each child is trained by 1 interventionist (usually an occupational therapist [OT] or physiotherapist). This therapy is based on MSL principles as the practice is structured (progressive increase of difficulties, segmenting the tasks, and movement abilities), intensive (high therapeutic dosage, high number of movement repetitions, and high motor engagement time), goal-oriented (functional goals set in collaboration with children and their families), “hands off” (ie, therapists adapt the therapeutic environment to allow voluntary motor control rather than guiding movements or passively inducing them), and motivating for the child (activities are fun, set as “games,” with always a positive reinforcement of task performance) (Figure 3). These rehabilitation games always involve bimanual use concomitant with postural request and stimulation of the lower extremities.

The therapeutic team is coached by trained supervisors (HABIT-ILE experts) who have previously set 5 functional goals for each participant at the end of the first assessment (T0) using the Canadian Occupational Performance Measure (COPM). Throughout the day and during daily team meetings, the supervision team adapts the therapeutic plan and the content of therapeutic sessions to ensure treatment specificity for each child.

##### HABIT-ILE@home (Treatment Group)

**Figure 4.**
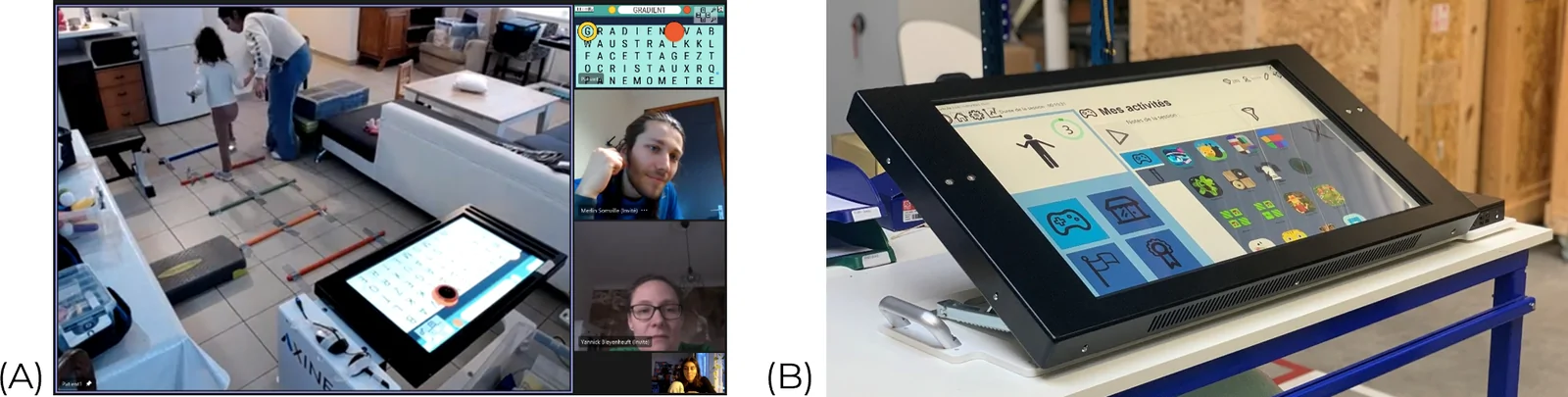
Home-based supervision and rehabilitation device. (A) Screenshot of a home supervision session during the pilot study. (B) REAtouch Lite rehabilitation device used for home-based training.

HABIT-ILE@home therapy will follow the same MSL principles as the on-site HABIT-ILE camp. The main differences will be the location of the therapy (ie, children’s home) and the person who will accompany the child and deliver the therapy (ie, caregivers who may vary during the therapy if needed). The role of the supervision team will remain crucial in providing remote coaching and guidance to plan the content of therapeutic sessions, adapt the therapeutic plan, and ensure treatment specificity for every child. As the therapeutic background cannot be similar between a caregiver and a therapist (OT and physiotherapist) usually involved in on-site camps, the supervision team will also play a role in coaching caregivers to observe movement abilities and disabilities, detect movement compensations, adapt the therapeutic environment, provide safety, and use positive reinforcement strategies (Figure 4A). To enable caregivers to perform these tasks, various resources will be provided by the supervision team. Before enrollment, families will receive a video detailing the intervention protocol in a child-friendly format, together with written information documents adapted for both adults and children. After enrollment and before the camp, a structured 1-hour training session divided into 2 parts will be given to caregivers. The first part will cover HABIT-ILE principles, including what constitutes bimanual activity and trunk and lower-extremity stimulation, goal setting, the “hands-off” approach, and intensity (high repetition, duration, and motor engagement). Task analysis and shaping will also be explained to help caregivers understand how activities are progressively adapted by the supervision team. Particular attention will be given to maintaining motivation through playful and meaningful activities. Caregivers will be trained on how to deliver feedback (timing, formulation, maintaining positive reinforcement, and avoiding information overload) and on safety (remaining close and vigilant without physically guiding the child to encourage active control while preventing falls). The second part will include theoretical and practical training on the REAtouch Lite device, including navigation of the interface, use of therapeutic games and communication tools, examples of exercises, object presentation and interaction with the screen, completion of activity logs, and daily organization of therapy. A detailed caregiver manual summarizing all elements will be provided. To further help and make the HABIT-ILE@home telerehabilitation program easier to implement, a telerehabilitation device will be delivered to every family. This telerehabilitation device will be the REAtouch Lite (Figure 4B; refer to Multimedia Appendix 1), a lighter and smaller version of REAtouch, a device that has already been used in previous HABIT-ILE on-site camps [24].

**Figure 5.**
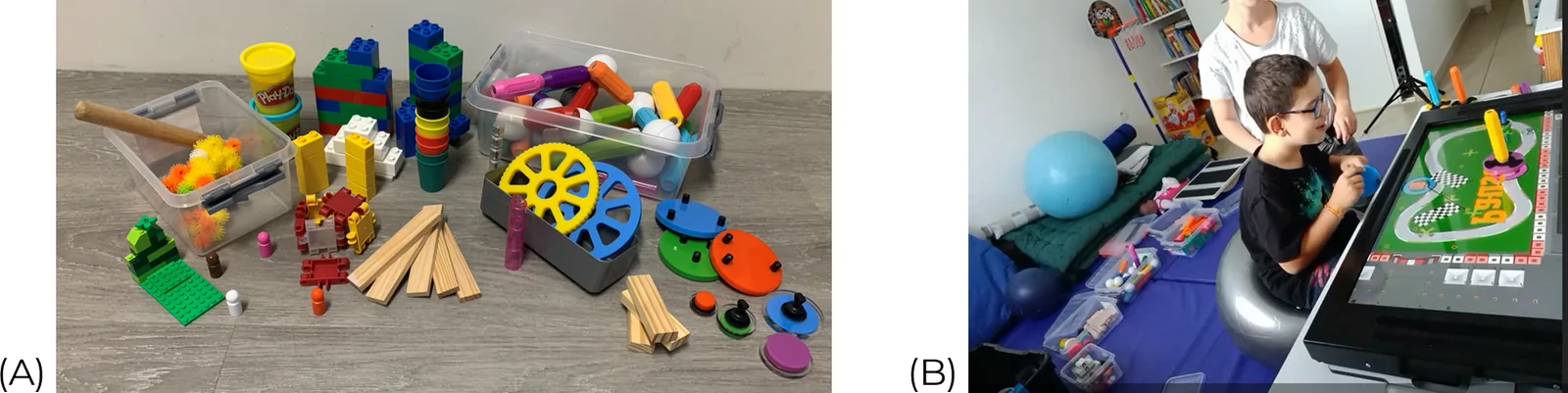
Materials and home therapeutic environment for HABIT-ILE@home therapy. (A) Set of objects made available to children during the 2 weeks of HABIT-ILE@home therapy. (B) Therapeutic environment at home.

The REAtouch Lite contains a wide range of rehabilitative games that can be individualized for each child and provides a visual environment to facilitate caregivers’ decision-making about how to apply MSL principles [24]. The REAtouch Lite is equipped with a telecommunications module for remote communications. In addition to the tablet, a mobile phone used as a microphone and camera and linked to the REAtouch Lite will be supplied. This mobile phone will be used by caregivers to take photos and videos and send them to the supervision team easily. This phone will be supplied with several phone holders, allowing for different camera angles during the supervision sessions. In addition, the mobile phone will enable caregivers to move the camera around the house and outside if required, depending on the functional goals selected. To allow the height of the screen to be adjusted (for postural control and lower-extremity involvement), the REAtouch Lite will also be accompanied by a height-adjustable table. In addition to the REAtouch Lite device, a set of therapeutically useful everyday objects and toys will be delivered to allow caregivers to adapt specifically to the functional goals set (Figure 5A). The therapeutic environment will also be enriched with additional equipment, including an adjustable bench, an inflatable ball, and/or a standing balance board depending on the child’s gross motor abilities and functional goals (Figure 5B).

**Figure 6.**
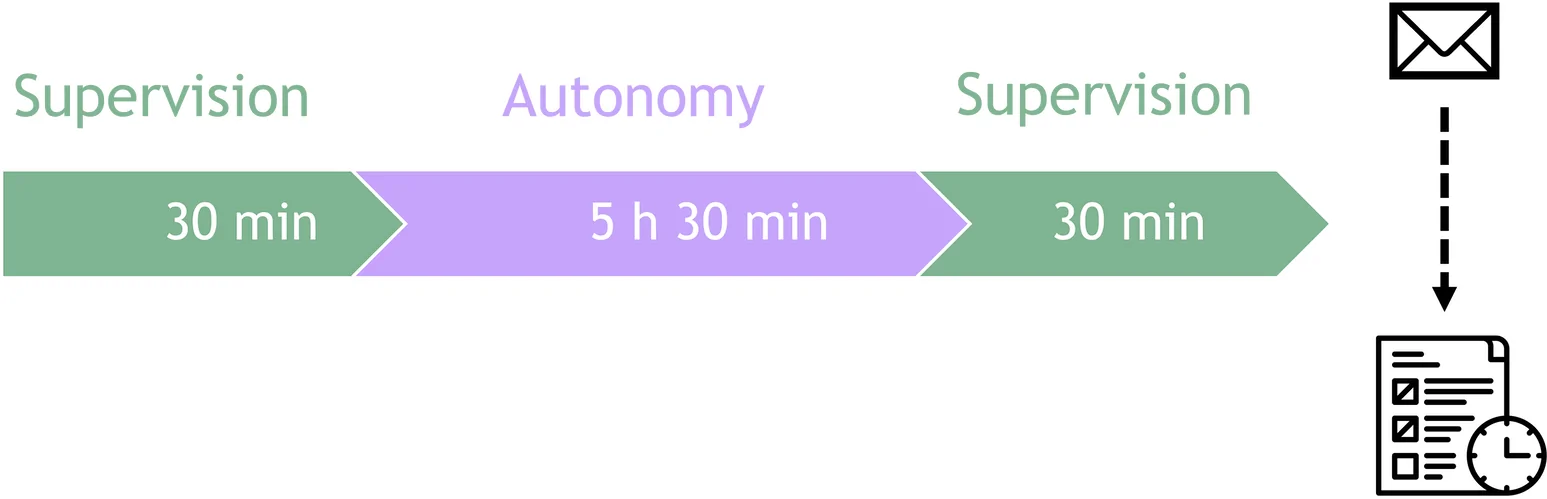
Course of a HABIT-ILE@home therapy day. A day of HABIT-ILE@home will begin with 30 minutes of remote supervision via video conferencing software, then the children and their caregivers will be left for 5 hours and 30 minutes in autonomy. Finally, the day will end with another 30 minutes of remote supervision. At the end of the day, the supervisor will send the treatment schedule for the next day.

To ensure gradual difficulty and specificity during the therapeutic games, 1 hour of remote supervision sessions per day will be planned within the daily 6.5 hours of therapy (30 min in the morning and 30 min at the end of the therapy day; Figure 6). Caregiver competency and fidelity to the intervention will be continuously monitored throughout the intervention. During the first 30-minute daily supervision session in the morning, supervisors will demonstrate how new activities should be implemented, provide guidance regarding task progression and feedback delivery, and ensure caregiver understanding of the proposed activities. During these supervision sessions, supervisors will also provide practical instructions regarding the type of objects to be used, the grasping patterns and positions to encourage, how objects should be presented to the child, and how games should be implemented. Supervisors will additionally answer questions from caregivers and children and provide support and solutions when difficulties arise.

During the second 30-minute supervision session at the end of the day, caregivers and children will demonstrate the activities performed throughout the intervention day, allowing supervisors to observe implementation, provide individualized feedback, address potential difficulties encountered during therapy, and support collaborative problem-solving strategies. Outside direct supervision sessions, caregivers will be encouraged to record and share videos of therapy sessions using the Axiphone (Axinesis) provided, enabling asynchronous monitoring and additional feedback. These daily interactions between supervisors and caregivers will allow close monitoring of the therapy dose completed and adherence to HABIT-ILE principles. At the end of each therapy day, supervisors will send caregivers a treatment schedule for the following day, including information regarding the type of objects to be used, grasping patterns, object orientation, types of games, and the estimated play time for each game (Figure 6). Fidelity will additionally be supported through individualized daily treatment schedules and activity logs documenting therapy dose and content.

### RCT2: Low- to Moderate-Dosage Home Follow-Up Interventions (9-Week HABIT-ILE@home Follow-Up vs Nonspecific Home Follow-Up)

#### Overview

For the RCT2, all children will take part in a low- to moderate-dosage home follow-up intervention, either with a specific HABIT-ILE@home follow-up or with a nonspecific home follow-up program managed by the children’s usual therapists. In both groups, the follow-up program will last 9 weeks, with a recommendation of 5 hours per week of therapy at home. The 45-hour follow-up dosage (5 h/wk for 9 wk) was selected to provide a feasible home-based intervention for families while remaining comparable to previous HABIT-ILE protocols and above dosage ranges previously associated with clinically meaningful motor improvements in children with CP [13,25]. The amount of therapy time actually spent by the children and the type of exercises actually performed will be documented using the same form in both groups, which families will be asked to complete weekly throughout the 9-week follow-up period. In cases where therapists do not wish to provide a specific home program, families will be offered a home exercise booklet developed by the Fondation Paralysie Cérébrale during the COVID-19 pandemic.

#### Nonspecific Home Follow-Up (Control Group)

For the 22 children who will receive the nonspecific follow-up at home in addition to their usual care, we will contact their usual therapists (OT or physiotherapist) and ask them to create a 5-hour weekly home program based on their usual practice for 9 weeks. This follow-up will correspond to heterogeneous therapist-driven interventions reflecting usual clinical practice, with no standardization of content imposed by the research team. This approach was chosen to reflect real-world clinical settings and the heterogeneity of rehabilitation practices between therapists, while ensuring a comparable amount of therapy time between the HABIT-ILE@home follow-up and nonspecific home follow-up groups.

#### HABIT-ILE@home Follow-Up (Treatment Group)

For the 22 children who will benefit from the HABIT-ILE@home follow-up in addition to their usual care, the treatment modalities will be the same as for the 2-week high-dosage HABIT-ILE@home intervention: it will be delivered at home, with 1 caregiver accompanying 1 child and the use of the REAtouch Lite. There will be 1-hour supervision session per week out of the 5 hours of therapy per week, adaptable according to the children’s and caregivers’ needs. A treatment schedule for 2 weeks will be sent at the beginning of every 2 weeks. The caregiver training, competency assessment, and fidelity monitoring procedures will be identical to those described for the high-dosage HABIT-ILE@home intervention, with the exception that direct remote supervision will be conducted for approximately 1 hour per week instead of daily.

### Data Monitoring Committee

At UCLouvain, there is no data monitoring committee for nondrug interventional studies. However, the Hospital-Faculty Ethics Committee of Saint Luc, composed of a majority of doctors (from UCLouvain or the Cliniques Universitaires de Saint-Luc), an unaffiliated general practitioner, 2 nurses, 3 pharmacists, 2 lawyers, 2 ethicists, 1 methodologist, 1 psychologist, 2 scientific collaborators, 3 patients’ representatives, and 2 healthy volunteers’ representatives, ensures that participants in clinical trials are not exposed to undue risks. The Committee reviews the study protocol and all relevant information regarding experimental treatment to ensure the safety of the research. It also verifies that the information provided to participants is presented in clear, accessible language, allowing them to give fully informed consent. This Ethics Committee operates independently from the study sponsor, with no members affiliated with the research project.

### Outcomes

#### Overview

The GMFM-66 will be defined as the primary outcome measure of the present study. The COPM will be considered a key secondary outcome, while all remaining secondary outcomes will be treated as exploratory measures.

#### Primary Outcomes

##### RCT1: High-Dosage Intervention (2-Week Home vs On-Site Camp)

The primary outcome measure for RCT1 will be the change in GMFM-66 observed after the 2 weeks of HABIT-ILE@home vs the HABIT-ILE on-site camp (T1-T0). The GMFM-66 evaluates children’s gross motor function skills through a large spectrum of tasks ranging from lying and rolling to walking, running, and jumping [26]. Linear measures (% of the full-range scale expressed in logits; from 0% to 100%) will be used to quantify clinical changes. Higher percentage values represent better gross motor function skills. The GMFM-66 item set version will be used as its responsiveness, reliability, and validity have been demonstrated in children with CP, including infants and toddlers [27,28]. The GMFM-66 assessments will be video-recorded and blindly assessed by trained examiners after study completion.

##### RCT2: Low- to Moderate-Dosage Home Follow-Up Interventions (9-Week HABIT-ILE@home Follow-Up vs Nonspecific Follow-Up)

The primary outcome is the same but will correspond to the change in GMFM-66 observed over the 9-week home follow-up period (HABIT-ILE@home follow-up vs nonspecific follow-up), measured from the end of the 2-week intensive intervention to the end of follow-up (T2-T1).

### Secondary Outcomes (RCT1 and RCT2)

#### Body Structures and Functions (Assessed at T0, T1, and T2)

Brain structural integrity will be evaluated using 3T brain MRI scanners. Prior to the scan, children will be familiarized with the MRI procedures. During the scan, they will watch an age-appropriate movie of their choice, except during the functional MRI acquisition. Structural brain images will be obtained using high-resolution 3D T1-weighted magnetization-prepared rapid acquisition with gradient echo and high-resolution 3D T2-weighted fluid-attenuated inversion recovery. Diffusion MRI data will be collected using a multishell approach with 20 directions at b=1000 s/mm², 60 directions at b=3000 s/mm², and 8 nondiffusion-weighted images (b=0 s/mm²). The diffusion acquisition will be divided into 4 blocks (22 directions/block) to facilitate efficient rescanning in the event of motion artifacts. Half of the blocks will be reverse phase-encoded to aid in correcting residual distortions caused by susceptibility inhomogeneities. Functional MRI data will be collected using a block design with an active hand and passive foot-tapping task. To correct susceptibility-induced distortions, 2 gradient-recalled echo images (TE1/TE2: 4.92/7.38 ms) will be used to acquire a field map for functional data. The total scan duration will be less than 1 hour.

Self-esteem of participants will be measured using the Self-ESTEEM-Kids-CP questionnaire [29]. This self-reported questionnaire covers various fields of self-esteem (global self-esteem, social acceptance, family relationship, friendship, scholastic competence, athletic competence, physical appearance, and behavior) and is illustrated in a comic strip-type format to make the assessment more fun and comprehensible for children. This tool, which is still under development, shows promising psychometric properties as reported in a preliminary study [29]. The linear measures are expressed as percentages of the full-range scale in logits, from 0% (low self-esteem) to 100% (high self-esteem).

Stereognosis will be assessed by the Improved Manual Form Perception Test [30,31], consisting of the tactile identification of concealed objects with one hand. Participants are required to identify the object as quickly as possible, either verbally or by pointing to the corresponding image on a standardized chart displaying multiple shapes. Both response time and the number of accurately identified objects will be recorded. It is reliable for school-aged children (aged 5‐18 years).

#### Activities (Assessed at T0, T1, and T2)

Bimanual performance will be assessed using the BoHA. It is a videotaped tool measuring how children with bilateral CP use both hands in bimanual activities. This tool also offers the opportunity to calculate an upper extremity asymmetry index. BoHA was validated for children with bilateral CP aged 18 months to 13 years [32]. For adolescents aged 14-18 years, the Adult Assisting Hand Assessment (Ad-AHA) can be administered using various bimanual activities, including a board game called Go with the Floe, which was the approach used in our study [33].

Unilateral gross manual dexterity of both hands will be evaluated by the Box and Block Test. This tool measures the number of blocks transported from one box to another in 1 minute. It is a valid and reliable test for children with mild to moderate bilateral manual ability impairments [34-36].

Unimanual dexterity in functional tasks will be measured using the Jebsen-Taylor Hand Function Test. This test records the speed required to perform 6 simulated everyday activities (turning over cards, picking up small objects, simulated eating, stacking checker pieces, and moving empty and full cans). The Jebsen-Taylor Hand Function Test is a valid test for children with mild to moderate unilateral manual ability impairments [37].

Manual performance, defined as the ability to manage daily activities requiring the use of the upper extremities (regardless of the strategies involved), will be measured with the ABILHAND-Kids questionnaire. Caregivers are asked to report on their child’s ease or difficulty in performing 21 manual activities. As a Rasch-built questionnaire, the linear measures correspond to the percentage of the full-range scale expressed in logits, from 0% (low manual performance) to 100% (high manual performance). ABILHAND-Kids is valid, reliable, unidimensional, and sensitive to change for children with CP [38,39].

Walk endurance will be evaluated by the 6-minute walk test. Participants are required to walk as far as possible along a 30-minute minimally trafficked corridor for a period of 6 minutes, with the measure being the 6-minute walk distance measured in meters. The 6-minute walk test is a reliable, reproducible, and valid tool for young ambulant people with CP [40-42].

Locomotor performance, defined as the child’s ability to move about effectively in the environment, will be measured by the ABILOCO-Kids questionnaire. Caregivers are asked to report on their child’s ease or difficulty in performing 10 locomotor activities. As a Rasch-built questionnaire, the linear measures correspond to percentages of the full-range scale expressed in logits, from 0% (low locomotor performance) to 100% (high locomotor performance). ABILOCO-Kids has been validated in children with CP [43].

Global activity performance, defined as the ability to manage daily activities requiring the use of the upper and/or lower extremities (regardless of the strategies involved), will be assessed by the ACTIVLIM-CP questionnaire. Caregivers are asked to report on their child’s ease or difficulty in performing 43 daily activities. As a Rasch-built questionnaire, the linear measures correspond to the percentage of the full-range scale expressed in logits, from 0% (low global performance) to 100% (high global performance). ACTIVLIM-CP is valid, reliable, unidimensional, and sensitive to change for children with CP [44,45].

Overall functional activity performance will be measured by the four domains of the Pediatric Evaluation of Disability Inventory–Computer Adaptive Test (PEDI-CAT): daily activities, mobility, social and cognitive activities, and responsibility. The PEDI-CAT includes a comprehensive item bank of 276 functional activities. A computer adaptive testing methodology is used to administer the tool by selecting the items that are most appropriate for children’s levels and minimizing the number of items required to ensure an accurate measure. The PEDI-CAT is designed for use with children and youth (birth through 20 years of age) with a variety of physical and/or behavioral conditions [46]. As a Rasch-built questionnaire, the linear measures correspond to the percentage of the full-range scale expressed in logits, from 0% (low manual performance) to 100% (high manual performance).

The children’s performance perceived by caregivers on functional goals and their satisfaction with the achieved performance will be quantified using the COPM [47]. Caregivers are asked to estimate their child’s performance and their satisfaction on a 10-point scale, from 1 (low performance or satisfaction) to 10 (high performance or satisfaction).

#### Participation

Participation, defined as children’s involvement in life situations (chosen because they make sense to them) in which they interact with others or which are linked to social roles, will be measured by the Participation In Life Situations–Kids–CP questionnaire. This self-reported questionnaire covers various domains of participation such as moving around in different places, recreation and leisure, school education, family relationships, interpersonal interaction, or problem solving. The life situations in the questionnaire are illustrated in a comic strip-type format to make the assessment more fun and comprehensible for children. This tool, which is still under development, will generate linear measures (percentage of the full-range scale expressed in logits), from 0% (low participation) to 100% (high participation).

#### Satisfaction Questionnaire (Feasibility and Adherence to Treatment)

A satisfaction questionnaire was developed to evaluate the feasibility of HABIT-ILE@home and compare it with the HABIT-ILE on-site method. Children’s and caregivers’ adherence to treatment will also be recorded and compared between both methods through the satisfaction questionnaire. The questionnaire will include items related to therapy burden, perceived feasibility, adherence, and acceptability of the intervention. It will be filled out by children, caregivers, and/or interventionists after the 2-week high-dosage intervention (T1) or after the 9-week home follow-up (T2).

#### Quantification and Qualification of Movements

The quantity and type of upper and lower extremity movements achieved during the 2-week interventions will be recorded using 3 accelerometers (2 placed on the dorsal side of the wrists and 1 on the upper side of the thigh, approximately in the middle of the thigh segment) to verify equivalence in therapy content between groups (2-wk HABIT-ILE@home vs HABIT-ILE on-site camp, RCT1). The Xsens DOT sensors will provide accurate measurements of accelerations and orientations, with demonstrated fair to excellent validity and reliability [48]. Triaxial acceleration data will be recorded over the 6.5 hours of daily therapy at a sampling frequency of 30 Hz. These data will be analyzed to yield an activity count (based on a predefined threshold for movement), an asymmetry ratio (comparing right and left upper extremity activity), and lower extremities activity positions (reflecting the percentage of time spent sitting, standing, or walking). These Inertial Measurement Unit (IMU) data will provide an objective measure of motor engagement and therapy intensity during the intervention.

### Statistical Analysis

#### Overview

Statistical analyses will be conducted following the collection of final measurements taken at the follow-up assessment. This will be conducted using SPSS software (IBM Corp; version 29.0.1.0), with a significance level set at *α*=.05.

All analyses will be conducted using an intention-to-treat approach, and missing data will be handled using multiple imputation methods.

Variables used for pairing (age, MACS, and GMFCS) were used to ensure baseline comparability between groups. Baseline characteristics, including sex, age, MACS, GMFCS, living area (urban or rural), type of housing, number of siblings, baseline outcome measures, and the type and amount of usual paramedical care (eg, physical therapy, occupational therapy, and psychomotor therapy) will be compared between groups to assess initial comparability using appropriate statistical tests (eg, Mann-Whitney rank sum test for continuous variables and Cochran-Armitage test for ordinal variables).

#### RCT1: High-Dosage Intervention (2-Week Home vs On-Site Camp)

Changes within each intervention group between baseline and post intervention (T1-T0) will first be assessed. Paired-sample *t* tests will be used when data are normally distributed, while the nonparametric Wilcoxon signed-rank test will be applied in the case of nonnormal distributions.

Noninferiority analyses will be performed by comparing changes obtained between groups (mean change in the HABIT-ILE@home group minus the HABIT-ILE on-site group). The mean obtained difference and its 95% CI will be compared to the prespecified NIM. The NIM has been set for each outcome measure based on the MCID.

For normally distributed data, the between-group difference and its CI will be estimated using an independent samples *t* test (or Welch *t*-test when variances are unequal). When the assumption of normality is not verified, Mann-Whitney *U* tests will be applied.

#### RCT2: Low- to Moderate-Dosage Home Follow-Up Interventions (9-Weeks HABIT-ILE@home Follow-Up vs Nonspecific Home Follow-Up)

Changes within each group during follow-up (T2-T1) will be assessed using paired-sample *t* tests when data are normally distributed, or Wilcoxon signed-rank tests in the case of nonnormal distributions.

The superiority analysis will be based on between-group comparisons using an analysis of covariance (ANCOVA) model. For each outcome, the model will be specified as outcome at T2 ~ outcome at T1 + RCT1_group × RCT2_group. This model will include the baseline value at T1 as a covariate and will test the main effects of the initial intervention (HABIT-ILE@home vs on-site), the follow-up intervention (HABIT-ILE@home follow-up vs nonspecific home follow-up), and their interaction. This approach will allow the effect of the second randomization (RCT2) to be evaluated while accounting for the initial allocation in RCT1.

The inference for superiority will be based on the main effect of RCT2_group (HABIT-ILE@home follow-up vs nonspecific home follow-up), adjusted for T1 values and RCT1_group. The interaction term (RCT1_group × RCT2_group) will be examined to explore whether the effect of the follow-up intervention differs depending on the initial intervention.

For normally distributed outcomes, model assumptions will be checked and standard ANCOVA estimates will be used. If normality is not verified, a nonparametric alternative based on rank-transformed ANCOVA will be applied.

### Ethical Considerations

The protocol is registered on ClinicalTrials.gov (NCT05740605) and was approved by the Hospital-Faculty Ethics Committee of Saint-Luc–UCLouvain, Belgium (clinical trial B4032022000142). All children’s parents will first receive information about the study protocol and will be asked to give written informed consent by signing the Information and Consent document for their child’s participation in the study. Children with bilateral CP will also be asked to provide assent after receiving age-appropriate information. All data will be collected and processed anonymously. Written consent for the use of data, including images and videos, was obtained from the participants, or from their parents or legal guardians in the case of minors.

## Results

Recruitment was completed in June 2024. Data collection was conducted from June 2023 to November 2024, and all interventions were completed by November 2024. Data analysis was completed in May 2025. Results are expected to be submitted in Fall or Winter 2026.

## Discussion

### Expectation for Main Findings

This protocol aims to develop and test a new way of delivering the HABIT-ILE intervention (HABIT-ILE@home; RCT1) and to assess the efficacy and benefits of including a specific HABIT-ILE@home follow-up treatment after high-dosage HABIT-ILE interventions (RCT2) in children with bilateral CP.

First, in RCT1, we hypothesize that children will demonstrate improvements following the HABIT-ILE@home intervention. We further hypothesize that improvements in the primary outcome measure, the GMFM-66, will be noninferior to those achieved after the conventional on-site HABIT-ILE intervention. In addition, we expect the HABIT-ILE@home intervention to be feasible to implement, with satisfactory levels of adherence.

Second, in RCT2, we hypothesize that children will demonstrate additional improvements following the HABIT-ILE@home follow-up intervention. We further hypothesize that children receiving a structured and specific HABIT-ILE@home follow-up will show greater improvements in the GMFM-66 compared to those receiving a nonspecific home follow-up. More specifically, we expect that children in the experimental group will demonstrate greater retention and transfer of motor and functional skills into daily life.

If these hypotheses are confirmed, HABIT-ILE@home could represent both an effective alternative to on-site intensive therapy and a valuable strategy to enhance long-term outcomes through structured follow-up.

### Comparison With Prior Work

Previous research supports the relevance of home-based, caregiver-delivered interventions to increase treatment intensity in children with CP. Regarding RCT1, Ferre et al [49] investigated a home-based Hand-Arm Bimanual Intensive Training (H-HABIT) program in young children with unilateral cerebral palsy (UCP; aged 2‐6 years), compared with a control group receiving an equally intensive lower extremity intervention (lower-extremity intensive functional training; LIFT) [49]. The H-HABIT group demonstrated greater improvements in manual dexterity, as measured by the Box and Block Test, and in functional goal performance (COPM-Performance), while no significant differences were observed for bimanual performance (assisting hand assessment; AHA). These findings suggest that home-based intensive bimanual training can effectively improve certain domains of upper limb function and goal-directed performance. However, this study was limited to unilateral CP, younger children, and did not include a full-body intervention such as HABIT-ILE.

Regarding RCT2, Hwang et al (2025) explored the added value of a home-based H-HABIT follow-up after modified constraint-induced movement therapy in children with UCP [50]. The experimental group, which received the structured home-based follow-up, showed greater improvements in bimanual performance (AHA), with a significant time × group interaction. In addition, caregiver feedback indicated high acceptability, with most participants reporting that the instructions were easy to follow and that remote interactions with therapists were beneficial. These results suggest that structured home-based follow-up interventions may help consolidate gains and potentially prevent posttreatment regression.

Taken together, these studies highlight the potential of home-based, family-centered approaches to enhance treatment intensity, accessibility, and continuity of care. However, they remain restricted to UCP populations and do not address MSL-based full-body interventions such as HABIT-ILE. The present study therefore extends prior work by applying these principles to children with bilateral cerebral palsy and by combining both an alternative delivery model (HABIT-ILE@home) and a structured postintensive follow-up approach.

### Strengths and Limitations

Despite its innovative approach, several challenges may arise in the implementation of this protocol.

One of the main difficulties will be related to caregivers’ ability to apply MSL principles effectively in a home setting. In RCT1, maintaining the child’s motivation for approximately 5.5 hours per day (excluding supervision), while managing fatigue during a 65-hour intensive program, may represent a substantial burden for families. The absence of a structured group environment and direct therapist supervision could further complicate adherence and engagement.

Regarding RCT2, integrating an additional 5 hours of therapy per week over a 9-week period into daily routines (including school, leisure, and family life) may also be challenging for some families, potentially affecting compliance and consistency.

However, this protocol also presents several important strengths. It addresses major barriers to accessing intensive rehabilitation, including geographical distance, financial constraints, and limited availability of trained therapists. It also provides an alternative for children who may not tolerate group-based interventions due to associated conditions such as autism spectrum disorder, attentional difficulties, hyperacusis, or exaggerated startle responses. Furthermore, it offers a potential solution to ensure continuity of care in situations such as pandemics or health care system constraints.

### Future Directions

If the findings of this study confirm the proposed hypotheses, HABIT-ILE@home could be implemented as a scalable and accessible model of intensive rehabilitation. This would support broader dissemination of MSL-based therapies beyond specialized centers and increase equity in access to care.

In addition, the integration of a structured home-based follow-up program could become a key component of HABIT-ILE interventions, with the aim of enhancing the transfer and long-term retention of functional gains in everyday life. Future work could focus on optimizing caregiver training, refining remote supervision strategies, and exploring digital tools to support adherence and engagement. It would also be important to identify which families and contexts are most suitable for this type of intervention, as well as to better understand the factors associated with improved adherence, participation, and successful implementation of home-based intensive rehabilitation programs.

## Supplementary material

10.2196/89872 Multimedia Appendix 1 Technical description of the REAtouch Lite device, associated therapeutic environment, and telecommunication tools supporting the delivery and supervision of the HABIT-ILE@home intervention.

10.2196/89872 Checklist 1 TIDieR-telehealth checklist.

## Abbreviations

Ad-AHA: Adult Assisting Hand Assessment
AHA: assisting hand assessment
ANCOVA: analysis of covariance
BoHA: Both Hands Assessment
COPM: Canadian Occupational Performance Measure
CP: cerebral palsy
GMFCS: Gross Motor Function Classification System
GMFM-66: Gross Motor Function Measure-66
H-HABIT: home-based Hand-Arm Bimanual Intensive Training
HABIT-ILE: Hand-Arm Bimanual Intensive Therapy Including Lower Extremities
IMU: Inertial Measurement Unit
LIFT: lower-extremity intensive functional training
MACS: Manual Ability Classification System
MCID: minimal clinically important difference
MRI: magnetic resonance imaging
MSL: motor skill learning
NIM: noninferiority margin
OT: occupational therapist
PEDI-CAT: Pediatric Evaluation of Disability Inventory–Computer Adaptive Test
RCT: randomized controlled trial
UCP: unilateral cerebral palsy
TIDieR: Template for the Intervention Description and Replication
