# Supplementary material for: HABIT-ILE@home for Children With Bilateral Cerebral Palsy: Protocol for Noninferiority and Superiority Randomized Controlled Trials
Source: JMIR Res Protoc. 2026 Aug 11;15:e89872. doi: 10.2196/89872 (PMC13460068; doi:10.2196/89872)
Supplement: Supplementary file 1 [file resprot-v15-e89872-s001.docx]

**The REAtouch®Lite device and therapeutic environment at home**

REAtouch®Lite description and its accompanying material

The REAtouch®Lite is a medical device created by Axinesis S.A. (Belgium) designed based on the previous development of the REAtouch® device. The REAtouch®Lite consists of a transportable interactive interface, equipped with a 32-inches (73 x 41cm) touch screen enabling interaction with tangible objects. It weighs 15 kilograms and measures 100 cm in length x 55 cm wide and 8 cm in height. The screen can tilt with an inclination of 0°/80° (Figure 1).

Aiming to notably allow telerehabilitation programs, the REAtouch®Lite is equipped with a telecommunication system, along with a dedicated therapeutic environment designed in collaboration with clinicians (Figure 1 & 2). The REAtouch®Lite comes with an easy-to-use/simplified smartphone, the “AXIphone®”, used as camera and microphone during teleconsultation, but also to allow participants to take picture and short movies during the intervention and send to the therapist supervising (Figure 1). Four phone holders (two suction cups, one large tripod and one portable tripod) are also supplied to allow easy and rapid adaptation of the viewpoint during telecommunications, as well as sessions outside of the home environment (with a 4G connection) (Figure 1).

a.

b.

**Figure 1.** REAtouch®Lite Hardware and telecommunication system

a. Inclinaison of the REAtouch®Lite screen
b. Portable tripod, large tripod and suction cups phone holder with AXIphone.

In addition to REAtouch®Lite, families were provided with a comprehensive therapeutic environment, including a variety of objects and toys designed for upper extremity training. The kit included five colored printed bases with Velcro fasteners and magnets, required to interact with the tablet in several REAtouch® games and allowing the attachment of objects tailored to individual grasping needs (Figure 2). The setup also featured an adjustable bench, a balance board, and an inflatable ball to support different seating positions based on the child’s motor abilities (Figure 2). Adjustable table legs (30–50 cm, 50-100cm, 60-130 cm) were supplied to modify the REAtouch®Lite's height for standing or sitting activities. An additional customization box was also provided with material such as adhesive Velcro, anti-slip sheets, painting tape, duct tape, and plastic bags… to adapt the equipment as needed.

a.

b. c.

**Figure 2.** Therapeutic environment provided for HABIT-ILE@home

a. Non-exhaustive contents of the object boxes
b. Participant during HABIT-ILE @home on a balance board. REAtouch®Lite is on the height-adjustable table legs
c. Participant during HABIT-ILE @home on an inflatable ball with all the material and boxes (an adjustable weight bench is also included with the therapeutic environment)

The REAtouch®Lite is equipped with software that enables telecommunication, remote screen control, and a variety of games and activities. The telecommunication system is running with Microsoft Teams, specifically adapted for home-based rehabilitation, allowing patients to interact directly with the supervision team displayed on the REAtouch®Lite screen (Figure 3 a). On the therapist's supervising side, in addition to viewing the patient through the AXIphone camera, the AnyDesk software can be used to remotely control the patient’s REAtouch®Lite screen (Figure 3 b).

Similar to the REAtouch®, the REAtouch®Lite is equipped with the TouchLAB software, offering a variety of virtual games and activities, including a medal-based reward system and virtual stores where participants can purchase items and customize their avatars. The games are programmable and customizable in terms of reaction time, difficulty, unimanual or bimanual modes, type of object interaction (specific or non-specific), and the use of dedicated REAtouch® colored printed bases.

a.

b.

**Figure 3.** Visuals and Examples of Telecommunication sessions

a. Overview of the REAtouch®Lite display screen during a supervision call
b. Overview of the screen of computer of the remote therapist supervising

The REAtouch®Lite promotes a wide range of object manipulations, including interactions with daily life objects, fully adaptable to each child’s profile, therapeutic goals, and motor/functional abilities. This flexibility allows therapists to implement structured and targeted interventions, particularly focusing on improving bimanual coordination of the upper extremities. The REAtouch® device was initially designed to provide an environment that facilitates therapist decision-making about how best structuring the intervention to target the application of motor skill learning principles while using a virtual device (1). A more precise description of the active role played by the REAtouch®Lite during the HABIT-ILE@home intervention will be displayed in Table 1.

Use of REAtouch®Lite in a HABIT-ILE@home context

The aim of this study protocol is to develop and test the efficacy of a home-based version of HABIT-ILE with the help of a caregiver accompanying the child. In this context, the REAtouch®Lite is planned to be used to ease the application of a HABIT-ILE@home intervention, notably thanks to its motivating environment and the opportunity to fully adapt to the patients’ need in terms of objects manipulations, plus height and tilt of the table/screen. In the context of HABIT-ILE@home, the REAtouch® is used for part of the therapeutic sessions. However, some of the therapy time is intentionally planned without the use of the REAtouch® to diversify the therapeutic environment and target activities/abilities that cannot be directly trained with the use of the device. On top of this, the REAtouch®Lite will be used as a telecommunication device, enabling to communicate with the families to provide remote demonstrations and live feedback during activities carried out at home and remote supervision sessions.

However, the REAtouch®Lite device itself, cannot promote the realization of a HABIT-ILE intervention and the whole principles of motor skill learning based interventions: intensity, structured practice, oriented towards self-determined functional goals, involving only voluntary motor control (hands-off), and motivating/fun. Moreover, during a HABIT-ILE intervention, therapeutic games/activities will always involve a bimanual activity as well as a postural demand of the trunk and simultaneous stimulation of the lower extremities. The REAtouch®Lite provides resources needed to implement these key elements, but without a trained supervision, nor caregivers, such key therapeutic principles cannot be implemented during REAtouch®-based sessions. The presence of an informed caregiver under the intermittent supervision of a trained therapist are crucial to ensure the correct application of a HABIT-ILE intervention and its principles of motor skill learning during home-based sessions. Table 2 details the application of motor learning principles during REAtouch®Lite sessions, and the role played by caregivers, supervision, and the REAtouch®Lite itself.

**Table 1.** Implementation of motor skill learning principles in HABIT-ILE@home with REAtouch®Lite

|  | REAtouch®Lite | Supervisors | Caregiver |
| --- | --- | --- | --- |
| **Intensity of practice** | +  *Games are developed to promote repetitions of screen -objects interactions, mainly integrating bimanual activities.* | ++  *By creating a complete schedule of 6.5 hours a day, containing activities that provide constant stimulation for the child (lower and upper extremities). By training/coaching caregivers to make the child active at least 70% of the therapy time.* | ++  *By accompanying their children during the 6.5 hours of activity per day* *and ensuring that the child performs a large number of repetitions of the trained abilities (high motor engagement time; >70% of therapeutic time).* |
| **Structured practice** | +/-  *By allowing an easily adaptable environment to allow therapist to adapt the program/activities based on children goals and abilities.* | ++  *Based on a task analysis, the supervisor analyzes the child's motor level and the abilities/skills to be trained to achieve de functional goals set. Choice of the trained abilities and the level of difficulty.* | +/-  *By applying what is suggested by the remote supervision therapist* |
| **Functional goal** | - | ++  *Functional goals are set children and parents during a semi-structured interview made with supervisor.*  *Choosing when to start practicing functional goals, and how to adapt the strategy when needed* | +  *Functional goals are set children and parents during a semi-structured interview made with supervisor.*  *Actively train the goals with children during training days.* |
| **Hands-Off** | +/-  *Does not provide assistance to movement.* | ++  *Provide daily life program with precise description of how to organize the therapeutic environment (type of objects, sitting/standing position, height of the table, etc) and the way to realize the activities to train the determined motor (or non-motor) abilities.*  *Provide feedback to the caregivers during remote supervision sessions.* | +  *Adapt the therapeutic environment and follow the instructions given by the supervision throughout remote supervision sessions and the daily program.* |
| **Fun/Motivation** | ++  *Owns a variety of fun games with direct and indirect rewards to motivate participants (medals and diamonds won during games, which can be used to buy items to customize the account / avatar in the interface).* | +  *Suggests activities and create games that will appeal to children's interests.* | ++  *Motivates children, knows their interests, plays with and against them all along the day.* |
| **Feedback** | - | ++  *Actively provide feedbacks during the remote sessions + coaching of parents about how to provide feedbacks during the whole intervention.* | ++  *Observes movements, gives feedback to the child with verbal guidance or demonstration to improve.* |

++ = play an active and essential role ; + = actively involved (play an active role for its application); ± = partially involved (allow its use, without playing an active role on it); - = not involved;

1.	Saussez G, Bailly R, Araneda R, Paradis J, Ebner-Karestinos D, Klöcker A, et al. Efficacy of integrating a semi-immersive virtual device in the HABIT-ILE intervention for children with unilateral cerebral palsy: a non-inferiority randomized controlled trial. J Neuroeng Rehabil. 2023;20(1):98.
