# Supplementary material for: HABIT-ILE@home for Children With Bilateral Cerebral Palsy: Protocol for Noninferiority and Superiority Randomized Controlled Trials
Source: JMIR Res Protoc. 2026 Aug 11;15:e89872. doi: 10.2196/89872 (PMC13460068; doi:10.2196/89872)
Supplement: Supplementary file 2 [file resprot-v15-e89872-s002.docx]

| **TIDieR-Telehealth item** | **Description in the present study** | **Where reported in manuscript** |
| --- | --- | --- |
| **1. Brief name** | The intervention is named **HABIT-ILE@home**, a home-based telerehabilitation adaptation of HABIT-ILE. | Title (page 1); Abstract (page 1); Introduction (page 2, line 1-50); Study interventions (page 10, line 158) |
| **2. Why** | HABIT-ILE@home was developed to improve access to intensive motor learning-based rehabilitation for children with bilateral cerebral palsy who may face geographical, organizational, financial, or family-related barriers to attending a two-week on-site camp. Remote delivery also aimed to support continuity of care and home transfer of skills. | Introduction (page 2, line 1-50); Discussion (page 24, line 475) |
| **3. Materials** | The intervention used the REAtouch® Lite telerehabilitation device, a tablet-based rehabilitation system with individualized games and remote communication features. A mobile phone with camera and microphone, phone holders, a height-adjustable table, therapeutic objects and toys, and postural/gross motor equipment such as an adjustable bench, inflatable ball, and/or standing balance board were provided according to the child’s needs. Caregivers also received a detailed intervention manual. | HABIT-ILE@home intervention section (page 10, line 158) |
| **4. Procedures** | HABIT-ILE@home followed the same motor skill learning principles as on-site HABIT-ILE: structured and intensive practice, progressive increase in task difficulty, task segmentation, functional goal-oriented activities, voluntary motor control, child motivation, and positive reinforcement. Activities involved bimanual use combined with postural and lower-extremity demands. Procedures were adapted from the on-site HABIT-ILE model to allow caregiver-delivered home practice with remote therapist supervision. | HABIT-ILE on-site; HABIT-ILE@home intervention section (page 10, line 158) |
| **5. Who provided** | The home-based intervention was delivered by a caregiver, such as a parent, family member, or relative. Caregivers received a one-hour training session before the intervention and were supported remotely by trained HABIT-ILE supervisors. The supervision team coached caregivers in observing motor abilities, detecting compensations, adapting the environment, ensuring safety, and using positive reinforcement. | HABIT-ILE@home intervention section (page 10, line 158) |
| **6. How** | The intervention was delivered at home using a telerehabilitation format. Caregivers accompanied directly the child, while therapists provided remote synchronous supervision through the REAtouch® Lite system and associated mobile phone/camera setup. Communication was bidirectional, allowing supervisors to observe the child, guide caregivers, answer questions, and adapt activities. | HABIT-ILE@home intervention section (page 10, line 158) |
| **7. Where** | The HABIT-ILE@home intervention took place in the child’s home. Remote supervision was provided by the trained supervision team. The comparator HABIT-ILE on-site camp took place in Belgium in a rehabilitation setting. | Study design (page 5, line 78); Study interventions (page 10, line 158) |
| **8. When and how much** | During RCT1, HABIT-ILE@home was delivered for 65 hours over two weeks, at a rate of 6.5 hours/day, 5 days/week. One hour of remote supervision was planned each day, divided into 30 minutes in the morning and 30 minutes at the end of the therapy day. During RCT2, the HABIT-ILE@home follow-up was delivered over 9 weeks, with a recommendation of 5 hours/week, including one hour of remote supervision per week. | Study design (page 5, line 78); RCT1 and RCT2 intervention sections (page 10, line 158) |
| **9. Tailoring** | The intervention was individualized according to each child’s functional goals, motor abilities, fatigue, motivation, and home environment. Five functional goals were identified using the COPM. Supervisors adapted activities, objects, grasps, positions, postural demands, and task difficulty throughout the intervention. Daily (RCT1) or biweekly (RCT2) treatment schedules were provided depending on the intervention phase. | HABIT-ILE@home intervention section (page 10, line 158); Outcomes - COPM (page 17, line 294) |
| **10. Modifications** | The intervention was designed to be adaptable to each child and caregiver context. Any adaptations concerned the therapeutic content, level of difficulty, activities, objects, positions, and treatment schedules, while maintaining the core principles of HABIT-ILE. No change in delivery mode from remote to in-person was planned in the protocol. | HABIT-ILE@home intervention section (page 10, line 158) |
| **11. How well – planned adherence/fidelity** | Treatment fidelity was planned to be supported through standardized caregiver training, a caregiver manual, daily remote supervision during the two-week intervention, weekly supervision during the follow-up, and individualized treatment schedules prepared by experienced HABIT-ILE supervisors. Adherence was planned to be monitored through caregiver-completed forms documenting the amount of therapy performed and the type of exercises completed. Satisfaction/adherence questionnaires were also administered. In addition, movement quantity and type during the two-week interventions were recorded using IMUs to help characterize and compare therapy content between groups. | HABIT-ILE@home intervention section (page 10, line 158); Satisfaction questionnaire (page 21, line 408) ; RCT2 intervention section (page 10, line 158); Quantification and qualification of movements (page 21, line 416) |
| **12. How well – actual adherence/fidelity** | As this manuscript reports the study protocol, actual adherence, fidelity, and intervention delivery will be reported in the future RCT results paper. |  |
